# Rapid Protein Extraction from Canola Meal Pre-Treated with Enzymatic Reactive Extrusion

**DOI:** 10.3390/foods15030498

**Published:** 2026-02-01

**Authors:** Sunandita Ghosh, Edith Cristina González Hernández, Xinmei Sha, Jeff Chow, Fernanda San Martin-Gonzalez, Qing Jin, Da Chen

**Affiliations:** 1 Department of Food Science, Purdue University, 745 Agriculture Mall Drive, West Lafayette, IN 47907, USA; 2 School of Food and Agriculture, University of Maine, Orono, ME 04469, USA

**Keywords:** thermal mechanical treatment, functionality, limited hydrolysis, plant proteins, amino acid composition, in vitro digestion

## Abstract

Conventional alkaline extraction of plant proteins typically requires highly alkaline conditions (pH ≥ 11) and extended extraction times (~1 h). Although protease addition can lower extraction pH and improve functionality, it often requires prolonged hydrolysis. In this study, enzymatic reactive extrusion (*eREX*) using Alcalase, followed by a short duration alkaline extraction (5 min, pH 9), was evaluated as an alternative approach for producing protein-rich extracts from canola meal. The *eREX* process increased protein recovery by 48% and 42% compared with alkaline extraction conducted without and with Alcalase, respectively. The resulting powdered extracts reached a protein content of up to 49% and consisted primarily of partially hydrolyzed proteins (10–23 kDa) with increased surface hydrophobicity. Amino acid analysis showed substantial enrichment of essential amino acids, particularly histidine and sulfur-containing amino acids. Functional properties were improved, including enhanced solubility across pH 2–10, high foaming stability (88%), and increased oil-binding capacity (~5.5 g g^−1^), while in vitro digestibility remained comparable (~85%). Techno-economic analysis indicated reductions in water use (~11%), energy consumption (~48%), and production cost (16–25%). Overall, *eREX* provides a rapid, higher-throughput, and cost-effective strategy for producing premium canola protein ingredients.

## 1. Introduction

Plant protein-based foods are, on average, two to four times more expensive than their animal-based counterparts [1]. Although improvements in formulation and processing are gradually reducing the price disparity, the gap persists. One major contributor to the high costs of plant-based foods is the plant protein ingredients themselves, which account for over 30% of total production costs [2]. Utilizing proteins derived from food and agricultural by-products has been proposed as a cost-effective strategy to reduce ingredient costs while promoting sustainability in food production [3].

Rapeseed is the second most produced oilseed after soybean with 90 million tons production in 2023–2024 [4]. Canola is a rapeseed cultivar that has lower antinutritional factors than rapeseed [5]. Canola meal, a by-product of oil extraction, contains 35–45% proteins [6,7] and is predominantly composed of the 11S globulin cruciferin and 2S albumin napin [8]. The proteins provide over 400 mg of essential amino acids per gram of protein, including tyrosine, cysteine, and methionine [8]. The latter two range from 40 to 49 mg g^−1^ protein, which position them superior to soy, pea, and wheat proteins in terms of amino acid profile.

Many approaches have been used to extract proteins from canola/rapeseed meal to produce isolate or concentrate, with alkaline–isoelectric point precipitation being widely adopted [5]. A pH value of 10–12 and mixing time of 0.5–1 h is commonly required to extract 40–70% proteins from canola/rapeseed meals [5,9]. The yield increases with the increase of pH value, but higher pH accelerates protein denaturation, uncontrolled hydrolysis, and lysinoalanine formation, resulting in diminished functionality and nutritional value [10]. Adding protease during alkaline extraction enables milder alkaline condition while raising protein recovery [11]. The resulting hydrolysates may also exhibit superior functionality such as solubility, oil absorption, emulsification, and foaming properties [10]. However, enzyme-assisted extraction requires 1–3 h hydrolysis time to achieve considerable yields [11]. It typically occurs in suspensions with low solid content (≤7%), leading to high energy consumption for proteolysis and enzyme inactivation (85–100 °C). Such prolonged hydrolysis also generates small peptides imparting bitterness.

To shorten the cycle time of alkaline-based and/or enzyme-assisted plant protein extraction, mass-transfer limitations should be addressed by accelerating protein diffusion into extraction medium and minimizing the lag between extraction and separation. Twin-screw extrusion emerges as a suitable intensification platform. Rotating screws within a heated, close-tolerance barrel delivers controlled shear, mixing, and efficient heat transfer, which fragments the feed and increases the interfacial area with the surrounding medium, thereby facilitating solubilization. This strategy is well documented in lignocellulosic biorefineries under alkaline or ionic-liquid conditions [12,13,14]. By contrast, extrusion has rarely been used as a stand-alone unit operation for direct plant protein extraction, aside from alfalfa leaves where in-barrel mechanical expression yields protein-containing juice [15,16]. This is mainly due to the short residence time of extrusion that inhibits sufficient alkaline solubilization of proteins from raw plant matrices.

Reducing the surface hydrophobicity and lowering the molecular weight of plant proteins promotes alkaline solubilization by increasing protein–water interactions. Limited proteolysis has been found to accomplish this in plant proteins [17]. When exogenous enzymes are dosed into extruder, known as enzymatic reactive extrusion (*eREX*), enzyme–substrate contact is intensified in high-solid matrix (≥20%), compressing reaction time from hours to minutes relative to stirred-tank hydrolysis [18,19]. *eREX* has been used for continuous production of protein hydrolysates with specified degrees of hydrolysis and high solubility/interfacial activity for high-protein beverages, emulsified foods, and clinical/sports nutrition [20,21,22]. However, applying *eREX* specifically to accelerate solubilization for plant-protein extraction, and thereby shortening extraction time and enhancing recovery yield, remains largely unexplored. By combining thermal–mechanical treatment and proteolysis, we hypothesize that *eREX* enables degrading plant proteins during the extrusion process to accelerate its diffusion under alkaline environment for rapid extraction.

In the present study, Alcalase was incorporated during extrusion of canola meal to facilitate partial protein hydrolysis, followed by a short-duration alkaline extraction. The protein recovery, physicochemical characteristics, and functional attributes, and the in vitro digestibility of the extracted canola proteins, were examined in comparison with alkaline extraction with and without Alcalase. A detailed techno-economic analysis was also conducted to quantify process efficiency and assess the feasibility of integrating this approach into larger-scale operations. The findings demonstrate enzymatic reactive extrusion could markedly shorten protein extraction time with a higher plant protein recovery, which potentially reduces production cost for wide incorporation in foods.

## 2. Materials and Methods

### 2.1. Materials

Solvent-extracted canola meal with 38.5% protein, 27.6% carbohydrate, 9.1% moisture, 5.9% ash, 1.2% phenol, and 17.7% others (lignin, etc.) was ground to pass through a 60-mesh screen prior to use. Alcalase (Catalog no: 126741, from *Bacillus licheniformis*, ≥0.75 Anson units/mL), bile bovine (B3883), pancreatin from porcine pancreas (P7545), and Pefabloc SC (76307) were purchased from Sigma Aldrich (St. Louis, MO, USA). Alpha amylase and pepsin were obtained from MP Biochemicals and Mallinckrodt AR (ACS), respectively. Mini-Protean TGX Stain-Free Precast Gels 4–20% (4568095), 10× Tris/Tricine/SDS running buffer (1610744), tricine sample buffer (1610739), Precision plus pre-stained protein standards of 2–250 kDa (1610377), and Bio-safe Coomassie Blue G-250 (1610787) were purchased from Bio-Rad Laboratories (Hercules, CA, USA). All other chemicals used were from Fisher Scientific (Waltham, MA, USA). Water used was deionized.

### 2.2. Extrusion Treatment

The extrusion process was carried out in a Brabender TwinLab-F 20/40 extruder. The equipment has two screws of 20 mm of diameter and 795 mm screw length. The extruder barrel temperature was set to 55 °C across the first four zones based on the optimum temperature of Alcalase [23]. The fifth zone (melting zone) was set to 110 °C to inactivate Alcalase. A round strand die head (100 mm × 125 mm) without nozzle was used at the end of the barrel for maintaining the diameter uniformity in the extrudates. The feed rate of canola meal was maintained at 2 kg/h and the screed speed was set at 200 rpm to balance the mixing and residence time. Alkaline water (pH 11) with Alcalase was injected through a pump with a moisture of 50%, 60%, and 65%. The Alcalase to dry canola meal ratio was maintained at 0.1% (*v*/*w*) based on our preliminary study (Figure S1). Canola meal without Alcalase were also extruded as control (Ex-Ctrl). The residence time was measured around 2.5 min and the pressure ranged between 20–25 psi.

### 2.3. Protein Extraction

Canola meal extrudates, with and without Alcalase treatment, were dispersed in water to a solids content of 10% (*w*/*v*), adjusted to pH 9.0, stirred for 5 min at ambient temperature, and centrifuged (15,000× *g*, 30 min) to obtain the supernatant fraction containing proteins. Non-extruded canola meal at the same solids content, with and without Alcalase, were also alkaline extracted at 55 °C to approximate the thermal conditions used during extrusion to facilitate Alcalase hydrolysis. Alcalase was added at 0.1% (*v*/*w*) enzyme to dry canola meal ratio, stirred for 5 min, and then heated at 100 °C for 3 min to inactivate the enzyme prior to centrifugation. The protein content of all supernatants was quantified using the modified Lowry protein assay, and protein recovery was calculated relative to the total protein present in the original canola meal. The extrudates with the highest protein recovery were chosen for further analysis. Protein extracts obtained from alkaline extraction of non-extruded canola meal, non-extruded canola meal with Alcalase, extruded canola meal, and extruded canola meal with Alcalase were designated as Ctrl, Ctrl-Alc, Ex-Ctrl, and Ex-Alc, respectively.

### 2.4. Proximate Analysis and Phenol Content

The proximate composition of the freeze-dried extract powder was determined following established protocols with slight modifications. Moisture content was assessed by heating the samples at 105 °C in an oven until a consistent weight was obtained. The ash content of the samples was determined in a muffle furnace at 550 °C for 20 h until a white–grey residue was obtained [24]. The total protein content was measured using the Kjeldahl method with a nitrogen-to-protein conversion factor of 5.7. The carbohydrates were extracted by a two-step hydrolyzing method as outlined by the National Renewable Energy Laboratory (NREL) [25] followed by determination using the phenol-sulfuric acid assay [24]. The lipid content was measured using the vanillin reagent method [26]. The total phenols in were determined by Folin Ciocalteu reagent phenols after extracting using 80% methanol [27]. The other components were determined by the percentage difference.

### 2.5. Characterization of the Proteins in Extract

#### 2.5.1. Degree of Hydrolysis

The degree of hydrolysis (DH) was calculated by using the *o*-phthalaldehyde (OPA) method [17]. Briefly, 5% suspensions of the extracts were mixed with OPA reagent for 2 min before measuring the absorbance at 340 nm. The complete hydrolysis of the proteins was also conducted by heating at 120 °C for 24 h under 6 N HCl. The DH was calculated as the ratio of the sample absorbance to that of complete hydrolysis and expressed in percentage.

#### 2.5.2. Molecular Weight Profile Determined by SDS-PAGE

The samples were dissolved in 1× Tris running buffer to obtain a final protein content of 4 mg mL^−1^. A 100 µL aliquot of these solutions was mixed with 95 µL tricine sample buffer and 5 µL of 2-Mercaptoethanol as the reducing agent. The mixture was heated in a boiling water bath for 10 min and then centrifuged (10,000× *g*, 15 min). The supernatants (10 µL) and marker (8 µL) were carefully loaded on to the 4–20% gels and electrophoresed at 200 V for 20 min. The gel was fixed, washed with water, stained with Coomassie blue and de-stained with dilute acetic acid before imaging using GelDoc Go Imaging System (Biorad, Hercules, CA, USA).

#### 2.5.3. Amino Acid Composition

The extracts (50 mg) were hydrolyzed with 10 mL of 6 N HCl containing 0.2 mL of 25 mM α-aminobutyric acid (AABA) as an internal standard and three drops of phenol. Samples were purged with nitrogen, sealed, and incubated at 105 °C for 24 h. After cooling, hydrolysates were diluted to 50 mL with distilled water, filtered through a 0.2 μm PTFE membrane, and freeze-dried. The dried samples were reconstituted in 1 mL of citric acid buffer (pH 2.2). Amino acid calibration standards (0–250 μmol/L) were prepared with 0.1 mM AABA as an internal standard. For derivatization, 10 µL of either standard or sample was mixed with 70 µL of AccQ•Fluor borate buffer and 20 µL of reconstituted AccQ•Fluor reagent, vortexed, and incubated at 55 °C for 10–15 min. Separation was performed on an Agilent 1200 HPLC system using an AccQ•Tag amino acid column (Nova-Pak C18, 4 µm, 150 × 3.9 mm, Waters) with a diode-array detector at 260 nm under a gradient elution of Eluent A (AccQ•Tag concentrate in water) and Eluent B (acetonitrile and 0.1% formic acid).

#### 2.5.4. Surface Hydrophobicity

The surface hydrophobicity (*H*_0_) was measured as described previously [28]. Samples were prepared at pH 7 to achieve various protein concentrations (0.01–0.1%, *w*/*v*), reacted with 2 mM 8-Anilino-1-naphthalenesulfonic acid (ANS) in the dark for 15 min before measuring fluorescence intensity at 390 nm excitation wavelengths of 390 nm and 480 nm and emission wavelengths. The *H*_0_ value for each sample was determined as the slope of the linear correlation between protein concentration and fluorescence intensity.

##### 2.5.5. ζ-Potential and Protein Solubility

The extract powders were dispersed in water to achieve a 1% (*w*/*v*) concentration. The pH of the suspensions was adjusted to 2–11 using either HCl or NaOH, stirred continuously at 25 °C for 30 min, and centrifuged at 10,000× *g* for 15 min. The ζ-potential of the above supernatants was determined using a Zetasizer (Malvern Panalytical, Worcestershire, UK) with a backscattering angle of 173° at 25 °C. The refraction index of protein used was 1.45. The protein content in the supernatants was measured by using modified Lowry Assay. The protein solubility was calculated as the ratio of the amount of the protein in the supernatant to the total protein content in the sample and expressed as percentage.

### 2.6. Functional Properties

#### 2.6.1. Foaming Capacity and Stability

The extracts (0.5 g) were mixed with 20 mL water using a homogenizer at 8000 rpm for 30 s. They were further homogenized at 10,000 rpm for 3 min in an ice bath. The volume of the foam formed was recorded for the foaming capacity (FC). To measure the foaming stability (FS) the volume of the foam remaining after 90 min was also recorded. The foaming capacity and stability of the samples were calculated using Equations (1) and (2) [29].

(1) $$\mathrm{Foaming}\;\mathrm{capacity}\;\left(FC\right)\;\left(\%\right)\;=\;\frac{Initial\;volume\;(\mathrm{mL})}{Volume\;of\;foam\;(\mathrm{mL})}\times 100$$

(2) $$\mathrm{Foaming}\;\mathrm{stability}\;(FS)\;(\%)\;=\;\frac{Initial\;volume\;(\mathrm{mL})}{Volume\;of\;foam\;after\;90\;\min\;(\mathrm{mL})}\times 100$$

#### 2.6.2. Water-Holding and Oil-Binding Capacity

The extracts (0.5 g) were mixed with water (5 g) and left to stand for 10 min and then vortexed for 10 sec [30]. This was repeated six times, then the extracts were centrifuged at 1000× *g* for 15 min. The pellet obtained was used to determine the water-holding capacity (WHC) using Equation (3). This process was repeated using oil instead of water to determine the oil-binding capacity (OBC).

(3) $$WHC\;or\;OBC\;(\%)\;=\;\frac{Weight\;of\;pellet\left(g\right)-Weight\;of\;sample\;(g)}{Weight\;of\;sample\;(g)}\times 100$$

#### 2.6.3. Emulsification Properties

The emulsification capacity (EC) was assessed based on the initial droplet size formed upon emulsification, while the ES was determined by monitoring changes in droplet size during 7 days of storage. Emulsions were made as per the procedure described in [29]. Briefly, extract suspensions of 1% (*w*/*v*) were prepared in pH 7 phosphate buffer (10 mM) containing 0.02% (*w*/*v*) sodium azide to prevent microbial growth. Pre-emulsions were prepared by homogenizing 3 mL soybean oil with 30 mL of protein suspension at 12,000 rpm for 1 min. Emulsification was carried out by further homogenization using a Nano DeBee high pressure homogenizer (BEE International, South Easton, MA, USA) at 5500 psi for 10 passes. The emulsions were collected by placing them in an ice bath and stored at 4 °C for further analysis. The emulsion mean-diameter (Z-Average) was measured on day 0 (fresh), 1, 3, and 7 using a Zetasizer (Malvern Panalytical, Worcestershire, UK) with a backscattering angle of 173° at 25 °C. The refractive index for protein used was 1.45.

#### 2.6.4. Gelation Dynamics

DHR-3 hybrid rheometer (TA Instruments, New Castle, DE, USA) was used to study the gelation dynamics at 15% (*w*/*v*) extract concentration. A 40 mm parallel plate geometry with a 1 mm gap was employed, and the geometry boundary was sealed with liquid paraffin to minimize evaporation. A temperature ramp from 25 °C to 90 °C at 5 °C/min was used, held at 90 °C for 30 min, and cooled to 25 °C at the same rate. The storage modulus (*G′*) and loss modulus (*G″*) were recorded throughout the process under 0.5% strain (within linear viscoelastic region) and 1 Hz frequency. At least triplicates were run for each sample.

### 2.7. In Vitro Digestion

In vitro digestion of the proteins was carried out following the INFOGEST protocol [31]. The enzymes, bile, and CaCl_2_ were freshly prepared before the experiment. The final enzyme activity of salivary amylase, pepsin, lipase, and pancreatin were 75 U mg^−1^, 2000 U mg^−1^, 60 U mg^−1^, and 100 U mg^−1^. The final concentration of bile used was 10 mM. Suspensions of extracts at 10% (*w*/*v*) were prepared and left overnight at 4 °C. To 2.5 mL of the sample, 2.5 mL of simulated salivary fluid was added and slowly stirred at 37 °C for 2 min. Simulated gastric fluid (5 mL) was added to this mixture and again slowly stirred at 37 °C for 2 h. The pH of the mixture was maintained at 3. Next, 10 mL simulated salivary fluid was added, the pH was maintained at 7 and allowed to sit for 2 h at 37 °C under slow stirring. To inactivate the enzymes, Pefabloc at 5 mM final concentration was added at the end of the digestion. The total soluble proteins were obtained from the supernatant after centrifugation (10,000× *g*, 15 min) of the digesta. The apparent digestibility of the proteins was determined by Equation (4).

(4) $$Apparent\;digestibility\;\left(\%\right)=\frac{Total\;soluble\;proteins\;after\;digestion}{Total\;proteins\;before\;digestion}\times 100$$

### 2.8. Techno-Economic Analysis

The TEA model was built using SuperPro Designer (Version 14, Intelligen, Inc., Scotch Plains, NJ, USA), in which mass and energy flows were tracked. The plant was designed to process 50,000 MT of canola meal per year, corresponding to a middle-sized plant. The production plant was scheduled to operate 330 days per year and 24 h per day. Equipment costs were obtained from previous models and the build-in cost in SuperPro Designer software (Version 14). Equipment costs were scaled to reflect differences in processing capacity using the customary exponential scaling relationship (Equation (5)). Costs were then updated to 2023 dollars using the Chemical Engineering Plant Cost Index (CEPCI), as shown in Equation (6):

(5) $${Cost}_{new}=\;{Cost}_{base}\times \;{\left({Size}_{new}/{Size}_{base}\right)}^{0.6}$$

(6) $${Cost}_{2023}={Cost}_{base}\times \left({CEPCI}_{2023}/{CEPCI}_{base}\right)$$

The total capital investment (TCI) was estimated as the sum of the fixed capital investment (FCI), working capital (assumed as 10% of FCI), and land cost (5% of FCI). The FCI comprises both direct expenses, such as equipment purchase, installation, instrumentation, piping, building structures, electrical systems, utilities, and site preparation, and indirect expenses, including construction overhead, engineering and supervision, contractor fees, legal costs, and contingencies. These categories were derived as standard percentage allocations relative to equipment cost. Table S1 summarizes the breakdown of variable and fixed operating expenses. Variable costs consist of chemicals, enzymes, utilities, and waste management, with values sourced from earlier studies (Table S1). Fixed operating expenses, such as labor, associated overhead, maintenance, insurance, and depreciation were estimated. After determining the total operating cost, the unit cost of protein production was calculated by dividing the annual operating cost by the yearly amount of recoverable protein product.

### 2.9. Statistical Analysis

The results were analysed using average and standard deviation of the measurements. All the experiments were carried out in either duplicates or triplicates. Statistical analysis was conducted by using analysis of variance (ANOVA) with the post hoc Tukey’s HSD significant difference test using OriginPro 2023b (Northampton, MA, USA). Significance was established at *p* < 0.05.

## 3. Results and Discussions

### 3.1. Extraction, Protein Yield, and Proximate Composition

**Figure 1:**
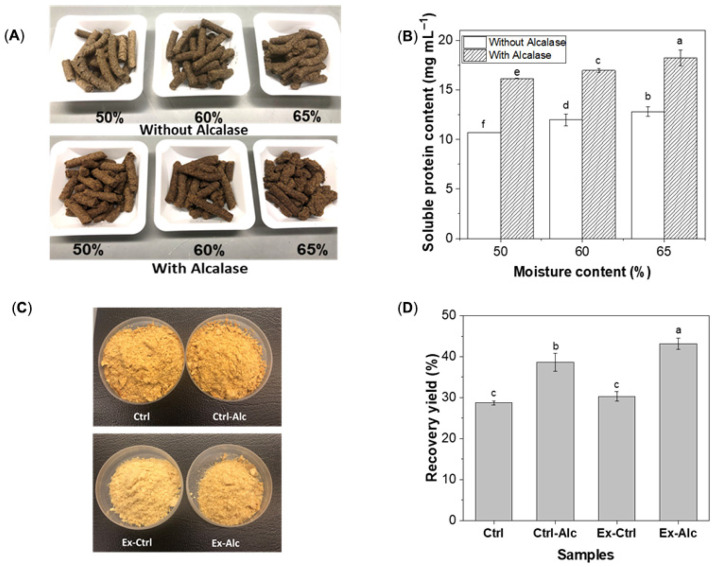
(**A**) Photographs of canola meal extrudates at different moisture content without and with Alcalase; (**B**) The soluble protein content of the extrudates at different moisture content; (**C**) Photographs of freeze-dried extracts; (**D**) Protein recovery yield of extracts. Ctrl, alkaline extraction without Alcalase; Ctrl-Alc, alkaline extraction with Alcalase; Ex-Ctrl, Alkaline extraction of extruded samples without Alcalase; Ex-Alc, alkaline extraction of extruded samples with Alcalase. Different letters on top of the bars indicate significant differences among the data (*p* < 0.05).

All the extrudates showed a uniform dark-brown color and cylindrical shape (Figure 1A). At 50% moisture, both extrudates (‘Without and With Alcalase’) exhibited surface fissures (Figure S2) due to limited plasticization. The shape intactness decreased with an increase in moisture. Following alkaline extraction, the soluble protein content in the extracts was higher for the extrudates treated with Alcalase, regardless of the initial moisture content (Figure 1B), with the highest value at 65% moisture. Increasing the moisture content could reduce canola meal matrix viscosity and enhance enzyme diffusion. It also promoted swelling of the dry meal particles, creating additional pores, and increasing the enzyme–protein contact surface. The elevated moisture levels could also accelerate protein hydration and facilitate its diffusion into water phase, thus facilitating protein extraction. Therefore, extrudate moisture at 65% was selected for subsequent analyses.

The extracts obtained from both non-extruded and extruded canola meal were freeze-dried. The extruded samples had a lighter brown color than their non-extruded counterparts (Figure 1C), possibly due to the partial degradation of pigments by extrusion [32]. Enzymatic hydrolysis (Ctrl-Alc) improved the protein recovery yield by approximately 35% compared to the untreated control (Ctrl) (Figure 1D). Whereas, extrusion alone (Ex-Ctrl) had no effect on the protein recovery yield compared to the Ctrl, the yield increased substantially, by ~50%, with Alcalase included (Ex-Alc), representing the highest protein recovery yield among all the extracts.

Proximate analysis of the extracts showed 49% proteins in Ex-Alc sample, which is significantly higher than those from Ctrl, Ctrl-Alc, and Ex-Ctrl samples (Table 1). The syneresis of proteolysis and extrusion-induced porosity and meal particle degradation (Figure S3) would enhance the alkaline solubility and diffusion [33], resulting in a higher protein purity in the extract. Carbohydrate levels remained relatively constant (27–28%) across all extracts. The carbohydrates are most likely to be pectins and hemicellulose, as they can be extracted by an alkaline medium. Increase in ash content was detected following extrusion, which may be attributed to the breakdown of matrix components that release minerals in the extractable phase [34]. The lipid content significantly dropped upon extrusion (both for Ex-Ctrl and Extr, With Alc). The “others” fraction ranged from 6–23% among the samples, which probably represents lignin. The value was the lowest in *eREX* extract due to the relatively increase of other components.

In conventional alkaline extraction, approximately 50–80% of the alkaline-soluble proteins precipitate during isoelectric precipitation (pH 4.5–5.5), while the remaining acid-soluble proteins are typically discarded unless recovered through membrane filtration. Although acid precipitation increases protein purity, it reduces overall recovery and elevates processing costs. In contrast, the *eREX* process omits the acid-precipitation step, producing a protein-rich extract that retains nearly all the alkaline soluble proteins and other valuable non-protein components such as carbohydrate, although by compromising protein purity. The developed protein extract is thus more applicable as protein concentrate rather than isolate. As shown in Figure S4, a 5 min extraction following *eREX* pretreatment achieved protein yields comparable to a 1 h extraction in conventional Alcalase-assisted processing, demonstrating the efficiency of the *eREX* process on facilitating protein extraction.

### 3.2. Characterization of the Proteins in Extract

#### 3.2.1. Degree of Hydrolysis and Molecular Weight

**Figure 2:**
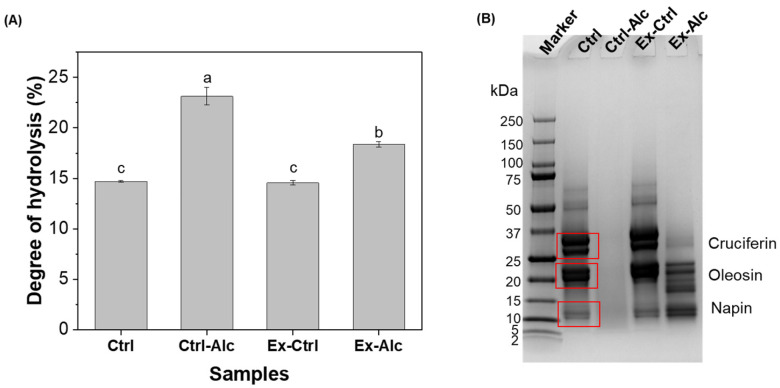
(**A**) Degree of Hydrolysis; (**B**) SDS profile of protein in the canola meal extracts. Ctrl, alkaline extraction without Alcalase; Ctrl-Alc, alkaline extraction with Alcalase; Ex-Ctrl, Alkaline extraction of extruded samples without Alcalase; Ex-Alc, alkaline extraction of extruded samples with Alcalase. Different letters on top of the bars indicate significant differences among the data (*p* < 0.05). The boxes highlight the corresponding proteins of the bands.

The proteins in Ctrl sample showed a DH of 15% (Figure 2A). A similar DH value (15%) was observed for Ex-Ctrl sample, suggesting that extrusion does not trigger peptide bond cleavage. Alcalase treatment (Ctrl-Alc) increased the DH to 23%, and *eREX* pretreatment (Ex-Alc) achieved a DH of 18%. A lower DH observed in Ex-Alc sample is probably due to a higher solid content that limited the diffusion of Alcalase into the canola meal for a lower hydrolysing efficiency. The OPA reagent used for DH measurement reacts with free amino groups; therefore, the measured absorbance reflects the abundance of these groups rather than directly quantifying the number of peptide bonds cleaved. The relatively high initial DH obtained using the OPA method likely reflects its sensitivity to trace levels of pre-existing low-molecular-weight peptides and free amino acids produced during the industrial oil extraction process [7], in addition to the inherent reactivity of lysine ε-amino groups. Nevertheless, the relative higher values of DH in Ex-Alc and Ctrl-Alc suggest an effective enzymatic hydrolysis is 3% and 8%, respectively.

The SDS-PAGE profile of the proteins in extract showed Ctrl sample had protein bands within 10–75 kDa (Figure 2B) with prominent ones located at around 30 kDa and 20 kDa corresponding to the α- and *β*-chains of cruciferin [35], and oleosins [8] respectively. Napin subunits were also detected at approximately 12 kDa [35]. Ex-Ctrl showed a profile comparable to the Ctrl sample, which agrees well with their similar DH values. Inclusion of Alcalase (Ex-Alc) generated peptide fragments primarily within the 10–23 kDa range indicate partial hydrolysis of the proteins. The bands were not visible with the appearance of a smear region in Ctrl-Alc sample, corresponding to a higher DH value.

#### 3.2.2. Amino Acid Composition

Amino acid profiles were analyzed to assess the nutritional attributes of proteins obtained from different extraction approaches (Table 2). The total amino acid content was lower than the protein content measured by Kjeldahl, which can be explained by methodological differences. The Kjeldahl assay quantifies total nitrogen and may overestimate protein content by up to 6% [36] due to the inclusion of non-protein nitrogen compounds, whereas amino acid analysis can underestimate total amino acids because tryptophan is destroyed and certain residues, such as lysine, cysteine, and methionine, undergo partial degradation during acid hydrolysis [37]. The total amino acid concentration increased from 241 mg g^−1^ extract in the Ctrl sample to 291 mg g^−1^ in Ctrl-Alc, and 386 mg g^−1^ in Ex-Alc. The substantial increase in total amino acids following *eREX* aligns with higher protein purity (Table 1).

Among individual amino acids, the most notable improvement was observed for histidine, which increased from 7.9 mg g^−1^ in Ctrl to 32 mg g^−1^ in Ex-Alc. Lysine content also increased from 3.5 mg g^−1^ to 5.6 mg g^−1^ exceeding that achieved by Alcalase treatment alone (Ctrl-Alc). This is consistent with previous reports indicating that partial proteolysis prior to or during thermal processing can reduce the extent of lysine loss by disrupting protein matrices and limiting Maillard-type condensation between lysine and reducing sugars [38]. The total sulfur-containing amino acids (cysteine + methionine) also increased from 9.2 mg g^−1^ in the Ctrl sample to 11.1 mg g^−1^ in Ctrl-Alc and 14.2 mg g^−1^ in Ex-Alc, which aligns with studies showing that enzymatic pre-treatment can enhance the release of sulfur-bearing residues that are otherwise partially inaccessible within protein structures [39]. The absence of reductions in any individual amino acid content indicates that *eREX* primarily enhanced amino acid availability rather than promoting thermal degradation or oxidation, consistent with previous study that high-moisture extrusion preserved heat-labile amino acids by reducing residence time under mild thermal conditions [40].

#### 3.2.3. Surface Hydrophobicity

**Figure 3:**
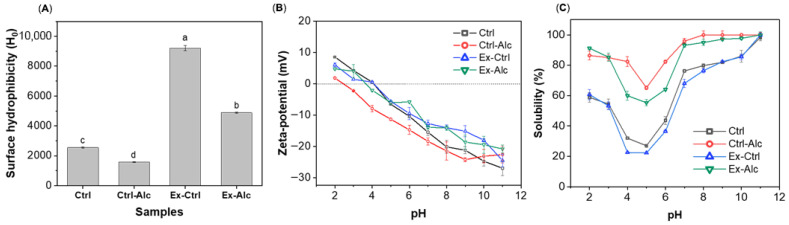
(**A**) Surface hydrophobicity. Different letters (on top of the bars) indicate significant differences among the data (*p* < 0.05); (**B**) Zeta-potential; (**C**) Solubility of protein extracts. Ctrl, alkaline extraction without Alcalase; Alc, alkaline extraction with Alcalase; Ex-Ctrl, Alkaline extraction of extruded samples without Alcalase; Ex-Alc, alkaline extraction of extruded samples with Alcalase.

The surface hydrophobicity (*H*_0_) of the extracts was determined to evaluate structural modifications induced by different extracting approaches. Extrusion (Ex-Ctrl) resulted in a significant increase in *H*_0_ compared to the Ctrl counterpart (Figure 3A), consistent with previous observations on soy proteins [41]. This increase can be attributed to the exposure of hydrophobic residues that were previously buried within the protein interior, due to molecular unfolding during extrusion. Alcalase hydrolysis (Ctrl-Alc) decreased the *H*_0_ compared to the Ctrl sample. Similar observations have been reported in pea hydrolysates obtained using Alcalase [17]. The *H*_0_ of Ex-Alc samples were higher than the Ctrl but lower than Ex-Ctrl. Alcalase is known to release peptides enriched in hydrophobic amino acids [42]. These peptides may undergo conformational rearrangement or aggregation during *eREX*, leading to the re-burying of the hydrophobic side chains and a relative increase in the surface hydrophilicity.

##### 3.2.4. ζ-Potential and Protein Solubility

Besides surface hydrophobicity, surface charge is another indicator that affects protein physiochemical properties and functionality. The ζ-potential values decreased with an increase in pH for all samples (Figure 3B). The Ctrl sample crossed zero around pH 4, which is consistent with the reported isoelectric point (pI) for rapeseed/canola proteins [43]. Alcalase hydrolysis (Ctrl-Alc) increased the ζ-potential values across most of the pH range (≤pH 10), consistent with proteolysis exposing ionizable residues and yielding peptides with more surface charge [44]. The Ex-Alc sample showed comparable ζ-potential values to that of Ex-Ctrl. For protein solubility, all samples showed a U-shape, with lower values between pH 4 and 6, and higher values in more acid or alkaline environments. This is within expectation, as plant proteins usually exhibit the minimal solubility at pH close to the isoelectric point (pH 4.5–5). When the pH is far away from isoelectric point, it becomes more protonated or deprotonated, exhibiting higher affinity to water. It must be noted that the ζ-potential did not show the same U-shape as the protein solubility. Since the protein extract contains other non-protein ingredients, such as cell wall polysaccharides, which contributed to the overall zeta potential values. Ex-Ctrl sample had the lowest protein solubility among the samples, consistent with its higher surface hydrophobicity (Figure 3A). The Ctrl-Alc and Ex-Alc samples significantly improved their solubility across the measured pH range. The improvement can be attributed to both reduction in molecular weight and increased exposure of ionizable groups after proteolysis [44].

### 3.3. Functional Properties of Extracts

#### 3.3.1. Foaming and Emulsification Properties

**Figure 4:**
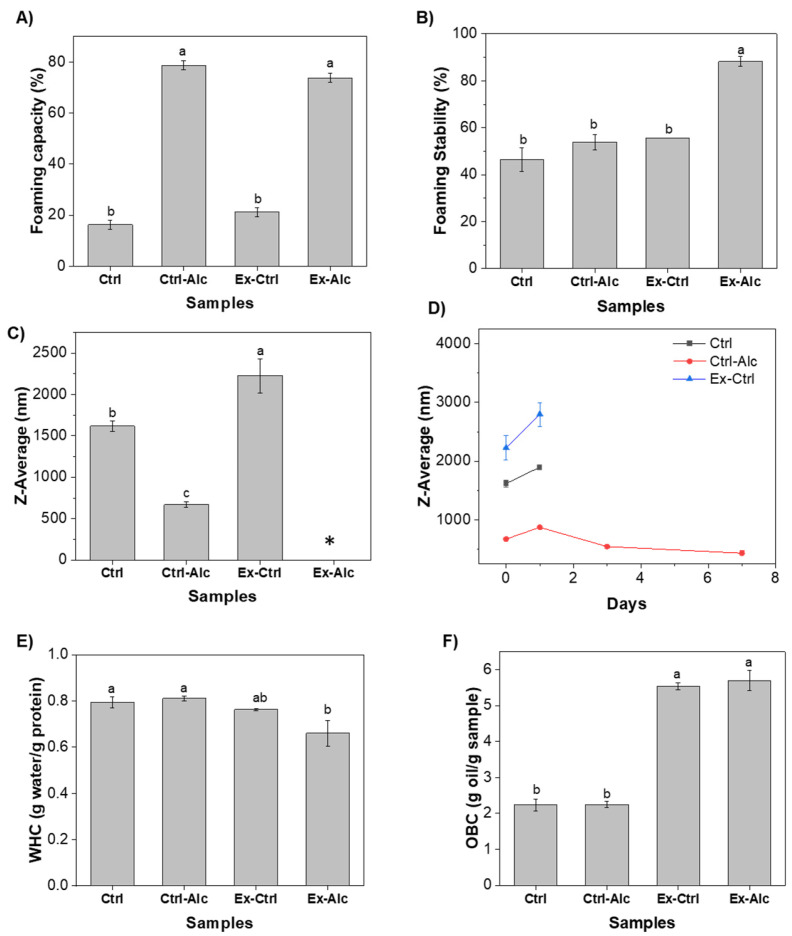
(**A**) Foaming capacity; (**B**) foaming stability; (**C**) emulsification capacity; (**D**) emulsification stability; (**E**) water-holding capacity (WHC); (**F**) oil-binding capacity (OBC) of canola meal extracts. Ctrl, alkaline extraction without Alcalase; Ctrl-Alc, alkaline extraction with Alcalase; Ex-Ctrl, alkaline extraction of extruded samples without Alcalase; Ex-Alc, alkaline extraction of extruded samples with Alcalase. Different letters on top of the bars indicate significant differences among the data (*p* < 0.05). (*) in (**C**) denotes droplet size was not obtained due to visible phase separation. In (**D**), by day 3 (for ‘Ctrl’ and ‘Ex-Ctrl’), no size data could be obtained due to visible phase separation.

The Ctrl sample showed a foaming capacity (FC) of 16%, which aligns with a previous reported value of 18% for rapeseed proteins (18%) [45]. Alcalase treatment significantly improved the FC to 75–80% in both the non-extruded (Ctrl-Alc) and extruded (Ex-Alc) extracts. Similar improvements were observed in Alcalase-hydrolyzed rapeseed proteins [45,46,47]. The FS was the highest for the Ex-Alc (88%), while the others exhibited significantly lower stability (~45–55%) (Figure 4B). This enhanced FC and FS suggests that Alcalase inclusion produced peptides that rapidly adsorb at the air–water interface and reduce the surface tension to sustain the foam [47].

Emulsions produced with Ctrl sample exhibited a Z-average of ~1600 nm, comparable to those stabilized with >0.5% (*w*/*w*) canola proteins (~1500 nm) [48]. Ctrl-Alc had significantly higher EC, shown as a smaller droplet size (~700 nm). Extrusion (Ex-Ctrl) diminished the EC, especially with the inclusion of Alcalase (Ex-Alc), for which the droplet size was not measurable due to the visual phase separation (Figures S5 and S6). Compared with intact proteins, medium-sized peptides generally exhibit superior emulsifying performance because they diffuse rapidly to the interface while retaining sufficient chain length and flexibility to form cohesive, viscoelastic oil–water interfacial films [49]. However, when these peptides associate into aggregates, as may occur during *eREX*, indicated by increased surface hydrophobicity, the resulting structures possess reduced mobility and limited ability to reorganize at the interface. Such aggregates are less effective in forming stable interfacial layers, leading to diminished EC [50]. The prepared emulsions were stored for 7 days to assess stability (ES). By day 1, all samples exhibited an increase in Z-average (Figure 4D), indicating the onset of flocculation [51]. By days 3 and 7, visible phase separation characterized by a serum and a cream layer was observed for Ctrl and Ex-Ctrl emulsions (Figure S4a,b). This confirmed progressive ES loss, consistent with destabilization initiated by flocculation, followed by creaming. In contrast, the Ctrl-Alc based emulsion showed a decrease in droplet size on day 3, indicating that the interfacial layer reorganized and re-equilibrated, thereby improving droplet stabilization [52].

#### 3.3.2. Water-Holding and Oil-Binding Capacity

The WHC of the Ctrl sample was ~0.8 g water g^−1^ extract (Figure 4E), which is lower than the values reported for rapeseed and canola proteins (1.6–3.9 g g^−1^) [53,54]. The relatively smaller value here may be attributed to the presence of non-protein constituents (such as phenolics, and minerals) that have a low capacity to retain water. The WHC was not affected by enzymatic treatment (Ctrl-Alc) or extrusion (Ex-Ctrl) treatment. However, combination of the two (Ex-Alc) slightly decreased the WHC (~0.65 g g^−1^) (Figure 4E). This reduction may be attributed to peptide aggregation during *eREX* that bury polar amino acid residues and reduce their water accessibility [54].

For the oil-binding capacity (OBC), the Ctrl sample exhibited a value of ~2.2 g oil g^−1^ extract, which is comparable to previously reported values for rapeseed and canola proteins (2.3–2.8 g g^−1^) [53,54]. Extrusion pretreatment led to a significant improvement in OBC for both ‘Ex-Ctrl’ and ‘Ex-Alc’ samples. This observation is consistent with findings for soybean [55] and hempseed [56] proteins, where extrusion increased the OBC by exposing hydrophobic groups that facilitate interactions with oil.

#### 3.3.3. Gelation Properties

**Figure 5:**
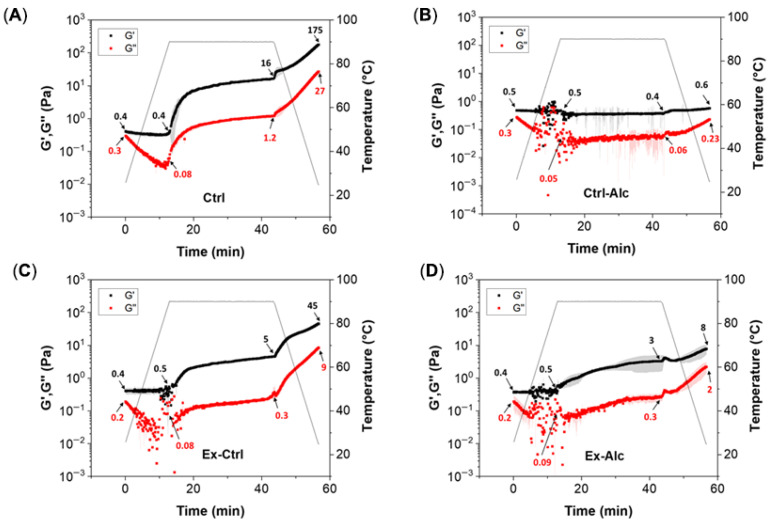
Gelation dynamics of canola meal extracts from different treatments. (**A**) Ctrl, alkaline extraction without Alcalase; (**B**) Ctrl-Alc, alkaline extraction with Alcalase; (**C**); Ex-Ctrl, alkaline extraction of extruded samples without Alcalase; (**D**) Ex-Alc, alkaline extraction of extruded samples with Alcalase. The number in the graphs indicates the *G′* and *G″* values at different heating and cooling stages.

Differences in structural characteristics and molecular size between untreated and treated canola proteins are expected to affect their gelling capacity during heat-induced gel formation. Changes in storage (*G′*) and loss modulus (*G″*) were thus recorded during the gelation process (Figure 5A–D). For all samples, *G′* values were slightly higher than *G″* prior to heating, indicating a weak gel nature of the extract suspension due to high solid content [28]. During the heating stage (25 °C to 90 °C), a modest decline in *G″* and relatively preserved *G′* was found. When the temperature was held at 90 °C, both *G′* and *G″* increased, except in the Ctrl-Alc sample. The Ctrl sample increased from 0.4 to 16 Pa, whereas only 2.5–4.5 Pa increment was observed in extruded samples (Ex-Ctrl, Ex-Alc). The magnitude of increment is mainly controlled by molecular interactions. Extrusion could denature proteins and form smaller aggregates which are usually less reactive than individual proteins after secondary heating. Upon cooling, *G′* showed a greater increase than during the heating stage in Ctrl, Ex-Ctrl, and Ex-Alc samples. Plant protein-based hydrogel is mainly dominated by non-covalent interactions [57]. At lower temperatures, more hydrogen bonding and electrostatic interactions are formed, thereby exhibiting higher moduli. Compared to Ctrl and Ex-Ctrl samples, the Ex-Alc sample had lower molecular weight, which may limit the intermolecular interactions and formation of continuous gel network, corresponding to a weaker gel. Based on the rheological study, *eREX* pre-treatment had an inferior effect on the gelling capacity of the protein extracts. It is noteworthy that the extracts studied here contained moderate amounts of carbohydrates, which may also be involved in the gelation process. As carbohydrate levels were comparable across all the extracts, their effects are likely similar and have limited contribution to the observed differences.

### 3.4. In Vitro Digestion

**Figure 6:**
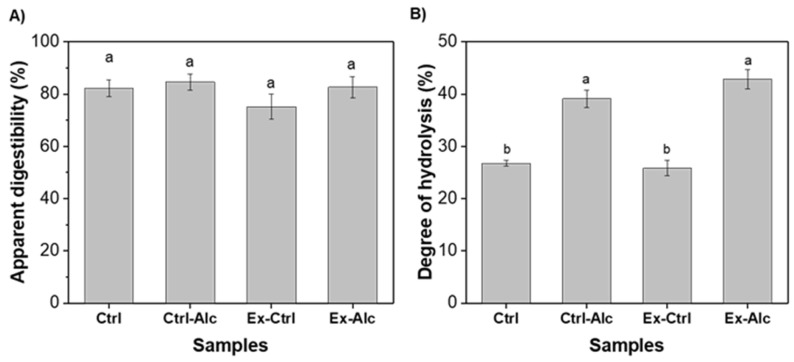
(**A**) Apparent digestibility and (**B**) degree of hydrolysis of in vitro digested extracts. Ctrl, alkaline extraction without Alcalase; Ctrl-Alc, alkaline extraction with Alcalase; Ex-Ctrl, Alkaline extraction of extruded samples without Alcalase; Ex-Alc, alkaline extraction of extruded samples with Alcalase. Different letters indicate significant differences among the data (*p* < 0.05).

The apparent protein digestibility of Ctrl sample was 85%. No significant differences were observed upon Alcalase treatment (Ex-Ctrl), extrusion (Ex-Ctrl), or combination of both. To further assess whether the extracts have undergone distinct degradation, the degree of hydrolysis (DH) of the digesta was measured simultaneously. The DH of the Ctrl sample was 27% (Figure 6B) and higher than the reported value of 19% for rapeseed meal [58]. The DH of the Ex-Ctrl sample showed no significant difference to that of Ctrl sample (Figure 6A), which agrees well with the digestibility values. The DH was significantly higher for both Ctrl-Alc and Ex-Alc, with similar values between the two. This enhancement can be attributed to the greater exposure and accessibility of cleavage sites to digestive enzymes resulting from Alcalase pre-treatment. However, this increase in DH did not translate into higher apparent digestibility (Figure 6A), a trend also observed for chickpea proteins subjected to enzymatic pretreatment [59]. DH measures the extent of peptide bond cleavage, while digestibility measures the distribution of peptides between soluble and insoluble phases, despite smaller peptides usually corresponding to higher solubility. However, when DH reaches high values, the produced peptides could aggregate and precipitate, becoming insoluble. The balance between the two determines the final solubility (digestibility).

### 3.5. Techno-Economic Analysis

To assess economic feasibility of large-scale production of protein concentrate using *eREX* method, a techno-economic analysis (TEA) of the process was conducted in comparison with alkaline extraction and enzyme-assisted alkaline extraction. Since extrusion-only (Ex-Ctrl) showed limited enhancement of protein recovery, it was excluded from the TEA. The total capital investment (TCI) for protein production from canola meal is summarized in Table 3. Ex-Alc shows the highest capital demand at approximately $16.4 million, largely driven by its higher equipment cost and correspondingly larger direct and indirect cost multipliers. Alkaline extraction (Ctrl) requires a moderate investment ($13.0 million), whereas Ex-Alc represents the lowest-capital option at about $10.9 million, reflecting its smaller equipment footprint and simplified processing configuration. Table 4 compares the annual operating costs and protein unit prices for alkaline extraction (Ctrl), enzymatic extraction (Ctrl-Alc), and reactive extrusion processing (Ex-Alc) plants, revealing substantial differences in economic performance among the three technologies. Ex-Alc demonstrates the lowest total operating cost ($33.2 million/year) and the lowest protein unit cost ($3.6/kg), driven primarily by reduced water consumption (due to high solid loading), lower chemical usage, and foamlower steam demand compared to the other processes. In contrast, Ctrl-Alc incurs the highest operating cost ($44.5 million/year), largely due to the high steam requirement (e.g., enzyme inactivation), reflected in utilities, contributing 44.9% of its total operating cost. Alkaline extraction shows a moderate cost profile ($39.6 million/year), but its heavy reliance on steam, large volumes of waste disposal, together with the lower protein yield keep its protein cost elevated at $10.6/kg. The findings from TEA demonstated the economic advantage of utilizing *eREX* on canola meal protein extraction and potentially reduced the production cost.

## 4. Conclusions

This study established a semi-continuous strategy for producing canola protein ingredients by integrating limited proteolysis during extrusion (*eREX*) with short-duration alkaline extraction. A key finding is that a 5 min extraction following *eREX* pre-treatment achieved protein recoveries comparable to those obtained with Alcalase-assisted extraction and greater than those from alkaline extraction alone, thereby reducing the extraction time from hours to minutes. Proteolysis and structural reorganization induced during *eREX* improved solubility, foaming stability, oil-binding capacity, and amino acid composition while maintaining high digestibility, although gelling capacity was reduced. Due to the short residence time and high solids content used during *eREX*, the process also substantially lowers water and energy consumption and decreases operating costs relative to conventional alkaline extraction. The estimated production cost and recommended selling price support the feasibility of scaling the process for industrial application. Because isoelectric precipitation was intentionally omitted, the resulting extract exhibited lower purity than a protein isolate. However, it is suitable as a protein concentrate that also delivers a moderate level of dietary fiber. Further optimization of extrusion conditions and enzyme dosage may enhance protein purity and recovery. A comprehensive assessment of alternative protease systems is recommended to evaluate their influence on process efficiency, composition, and functional performance of the extracted proteins from canola meal and other oilseed materials.

## Figures and Tables

**Table 1:** Proximate analysis (%) of freeze-dried canola meal extracts. Ctrl, alkaline extraction without Alcalase; Ctrl-Alc, alkaline extraction with Alcalase; Ex-Ctrl, alkaline extraction of extruded samples without Alcalase; Ex-Alc, alkaline extraction of extruded samples with Alcalase. ‘Others’ is primarily composed of acid-insoluble lignin. Different superscript letters indicate significant differences among the data (*p* < 0.05).

Samples	Moisture	Ash	Protein	Carbohydrate	Lipid	Others	Phenol
Ctrl	4.6 ± 0.0 ^b^	10.8 ± 0.2 ^b^	31.4 ± 0.1 ^c^	27.8 ± 0.2 ^a^	0.13 ± 0.01 ^b^	22.5 ± 0.3 ^a^	2.6 ± 0.1 ^b^
Ctrl-Alc	5.3 ± 0.2 ^a^	10.7 ± 0.2 ^b^	37.9 ± 0.1 ^b^	28.2 ± 0.6 ^a^	0.17 ± 0.00 ^a^	14.9 ± 0.7 ^b^	2.8 ± 0.1 ^ab^
Ex-Ctrl	2.7 ± 0.1 ^d^	12.5 ± 0.2 ^a^	32.1 ± 0.8 ^c^	27.5 ± 0.0 ^a^	0.04 ± 0.00 ^c^	22.7 ± 0.9 ^a^	2.4 ± 0.0 ^c^
Ex-Alc	3.6 ± 0.2 ^c^	11.8 ± 0.2 ^a^	49.2 ± 0.7 ^a^	26.6 ± 1.1 ^b^	0.03 ± 0.00 ^c^	6.0 ± 1.3 ^c^	2.8 ± 0.0 ^a^

**Table 2:** Amino acid composition of extracts from canola meal. Ctrl, alkaline extraction without Alcalase; Ctrl-Alc, alkaline extraction with Alcalase; Ex-Ctrl, Alkaline extraction of extruded samples without Alcalase; Ex-Alc, alkaline extraction of extruded samples with Alcalase. AA (amino acid); Asx (asparagine + aspartic acid) and Glx (glutamine + glutamic acid). The data were expressed as mean ± SD. Different superscript letters within the same row indicate significant differences among the data (*p* < 0.05).

Amino Acid (mg g^−1^ Extract)	Ctrl	Ctrl-Alc	Ex-Ctrl	Ex-Alc
Essential AA				
His	7.9 ± 0.4 ^cd^	9.3 ± 0.9 ^c^	17.8 ± 2.7 ^b^	31.7 ± 2.0 ^a^
Ile	7.6 ± 0.6 ^b^	7.2 ± 0.4 ^b^	6.4 ± 0.9 ^b^	10.7 ± 0.2 ^a^
Leu	14.0 ± 1.3 ^b^	15.1 ± 0.9 ^b^	12.5 ± 1.1 ^b^	20.2 ± 0.3 ^a^
Lys	3.5 ± 0.3 ^c^	4.5 ± 0.3 ^b^	2.5 ± 0.2 ^d^	5.6 ± 0.4 ^a^
Met	5.2 ± 0.4 ^c^	6.5 ± 0.4 ^b^	4.4 ± 0.4 ^c^	7.8 ± 0.2 ^a^
Phe	9.1 ± 0.8 ^a^	8.0 ± 0.5 ^a^	8.2 ± 0.6 ^a^	11.4 ± 0.2 ^a^
Val	6.0 ± 0.5 ^c^	9.4 ± 0.6 ^b^	5.8 ± 0.5 ^c^	12.1 ± 0.2 ^a^
Thr	8.4 ± 0.6 ^c^	11.6 ± 0.6 ^b^	7.4 ± 0.4 ^c^	13.8 ± 0.1 ^a^
Total essential AA	61.2 ± 1.1 ^c^	71.6 ± 1.0 ^b^	65.1 ± 1.8 ^c^	113.3 ± 1.2 ^a^
Non-essential AA				
Ala	8.6 ± 0.6 ^c^	11.2 ± 0.7 ^b^	7.9 ± 0.4 ^c^	14.2 ± 0.6 ^a^
Arg	9.5 ± 1.2 ^a^	10.8 ± 0.6 ^a^	7.5 ± 0.7 ^a^	12.9 ± 0.5 ^a^
Asx	56.0 ± 3.8 ^bc^	61.5 ± 3.4 ^b^	50.4 ± 1.3 ^c^	78.8 ± 1.3 ^a^
Cys	4.0 ± 0.3 ^b^	4.7 ± 0.3 ^ab^	3.8 ± 0.2 ^b^	6.3 ± 0.2 ^a^
Glx	12.2 ± 0.9 ^b^	12.1 ± 0.7 ^b^	11.1 ± 0.9 ^b^	17.9 ± 0.4 ^a^
Gly	7.6 ± 0.6 ^bc^	9.0 ± 0.4 ^b^	6.6 ± 0.4 ^c^	11.2 ± 0.2 ^a^
Pro	12.6 ± 0.7 ^c^	16.9 ± 0.8 ^b^	10.8 ± 0.6 ^c^	20.2 ± 0.7 ^a^
Ser	56.7 ± 5.4 ^b^	78.5 ± 7.5 ^a^	52.3 ± 5.4 ^b^	91.22 ± 10.9 ^a^
Tyr	12.7 ± 1.1 ^b^	14.9 ± 0.9 ^b^	11.3 ± 0.9 ^b^	20.1 ± 0.3 ^a^
Total Non-essential AA	179.9 ± 4.0 ^c^	219.5 ± 4.9 ^b^	161.6 ± 3.4 ^d^	272.8 ± 6.4 ^a^
Total AA	241.1 ± 4.2 ^c^	291.1 ± 4.9 ^b^	226.6 ± 3.8 ^c^	386.0 ± 6.5 ^a^

**Table 3:** Total capital investment ($) for alkaline extraction (Ctrl), enzymatic extraction (Ctrl-Alc), and enzymatic reactive extrusion (Ex-Alc) processing plants.

Item	Ctrl ($)	Enzyme ($) Extraction	Extrusion ($) Extraction
Purchased equipment cost (E)	2,598,747	3,291,207	2,187,851
Purchased-equipment installation (39% E)	1,013,511	1,283,571	853,262
Instrument and controls (26% E)	675,674	855,714	568,841
Piping (31% E)	805,611	1,020,274	678,234
Electrical systems (10% E)	259,875	329,121	218,785
Buildings (29% E)	753,637	954,450	634,477
Yard improvements (12% E)	311,850	394,945	262,542
Service facilities (55% E)	1,429,311	1,810,164	1,203,318
**Total direct plant costs (302% E)**	7,848,215	9,939,445	6,607,310
Engineering and supervision (33% E)	857,586	1,086,098	721,991
Construction expenses (39% E)	1,013,511	1,283,571	853,262
Legal expenses (4% E)	103,950	131,648	87,514
Contractor’s fee (17% E)	441,787	559,505	371,935
Contingency (35% E)	909,561	1,151,922	765,748
**Total indirect costs** (**128% E**)	3,326,396	4,212,745	2,800,449
Fixed capital investment (FCI, 430% E)	11,174,611	14,152,190	9,407,760
Working capital (10% of FCI)	1,240,382	1,570,893	1,044,261
Land (5% of FCI)	558,731	707,610	470,388
**Total capital investment**	12,973,723	16,430,693	10,922,409

**Table 4:** Annual usage, operating cost, and protein unit price of alkaline extraction (Ctrl), enzymatic extraction (Ctrl-Alc), and enzymatic reactive extrusion (Ex-Alc) processing plants.

Parameters	Alkaline Extraction		Enzymatic Extraction		Reactive Extrusion Extraction	
	Annual Use	Annual Cost ($)	Annual Use	Annual Cost ($)	Annual Use	Annual Cost ($)
**Raw materials**						
Canola meal (kg)	50,000,000	15,000,000	50,000,000	15,000,000	50,000,000	15,000,000
Alcalase (kg)	-	-	50,000	1,150,000	50,000	1,150,000
HCl (kg)	180,576	39,727	180,576	39,727	129,888	28,575
NaOH (kg)	220,000	99,000	220,000	99,000	182,000	81,900
Water (ton)	445,885	312,120	445,885	312,120	195,385	136,770
*Sub-total raw materials*		15,450,846 (43.6%) ^a^		16,600,846 (37.9%)		16,397,245 (55.6%)
**Waste discharge**						
Solid waste disposal (ton)	103,942,000	6,236,520	101,257,000	6,075,420	92,528,900	5,551,734
*Sub-total waste discharge*		6,236,520 (17.6%)		6,075,420 (13.9%)		5,551,734 (18.8%)
**Utilities**						
Electricity (kWh)	9,844,677	639,904	9,908,078	644,025	31,997,512	2,079,838
Steam (MT)	695,229	11,818,893	756,463	12,859,871	253,832	4,315,144
Cooling water (MT)	-	-	6,176,391	6,176,391	-	-
*Sub-total utilities*		12,458,797 (35.2%)		19,680,287 (44.9%)		6,394,982 (21.7%)
**Labor**						
Total salaries		300,000		300,000		300,000
Labor burden		270,000		270,000		270,000
*Sub-total labor*		570,000 (1.6%)		570,000 (1.3%)		570,000 (1.9%)
**Other overhead**						
Maintenance		77,962		98,736		52.905
Property insurance		78,222		99,065		53.081
*Sub-total other overhead*		156,185 (0.4%)		197,802 (0.5%)		105,985 (0.4%)
**Depreciation**		558,731 (1.6%)		707,610 (1.6%)		470,388 (1.6%)
**Total operating cost**		39,623,239 (100%)		44,452,575 (100%)		33,182,341 (100%)
**Unit cost of protein**		$10.6/kg		$7.0/kg		$3.6/kg

^a^ Number in parenthesis represents the total operating cost share of each category.

## Data Availability

The original contributions presented in this study are included in the article/Supplementary Material. Further inquiries can be directed to the corresponding author.

## Supplementary Materials

The following supporting information can be downloaded at: https://www.mdpi.com/article/10.3390/foods15030498/s1, Table S1. Operating costs applied in the study. Figure S1. SDS-PAGE profile of canola meal proteins extracted with varying Alcalase concentration. Figure S2. Enlarged photographs of canola meal extrudates at 55% moisture content without and with Alcalase. Figure S3. SEM micrographs of canola meal extracts of untreated control (Ctrl) and enzymatic reactive extrudates (Ex-Alc) at 65% moisture. Figure S4. Soluble protein content of non-extruded controls (Ctrl) and extrudates (Extr) at 5, 60, and 120 min of alkaline extraction at pH 9, both without and with Alcalase. Figure S5. Photographs of emulsions formed with canola meal extracts at (A) day 0 (B) day 1. Figure S6. Photographs of emulsions formed with canola meal extracts at (A) day 3 (B) day 7. Reference [60] is cited in the Supplementary Materials.

## Abbreviations

The following abbreviations are used in this manuscript:

Alc	Alcalase
Ctrl	Control
Ctrl-Alc	Protein extract from Alcalase-assisted extraction
DH	Degree of hydrolysis
EC	Emulsion capacity
ES	Emulsion stability
Ex-Ctrl	Protein extract from extruded canola meal
Ex-Alc	Protein extract from extruded canola meal containing Alcalase
FC	Foaming capacity
FS	Foaming stability
H_0_	Surface hydrophobicity
pI	Isoelectric point
SDS-PAGE	Sodium dodecyl sulfate–polyacrylamide gel electrophoresis
TEA	Technoeconomic analysis
TCI	Total capital investment
WHC	Water-holding capacity
OBC	Oil-binding capacity
