# Supplementary material for: Rapid Protein Extraction from Canola Meal Pre-Treated with Enzymatic Reactive Extrusion
Source: Foods. 2026 Feb 1;15(3):498. doi: 10.3390/foods15030498 (PMC12896430; doi:10.3390/foods15030498)
Supplement: Supplementary file 1 [file foods-15-00498-s001.zip › foods-3991253-supplementary.pdf]

Rapid protein extraction from canola meal pre-treated with enzymatic reactive extrusion

Sunandita Ghosh [1], Edith Cristina González Hernández [1], Xinmei Sha[1], Jeff Chow [1], Fernanda San Martin-Gonzalez [1], Qing Jin[2], and Da Chen [1,*]

[1]Department of Food Science, Purdue University, 745 Agriculture Mall Drive, West Lafayette, IN 47907, USA; ghosh197@purdue.edu (S.G.); shax@purdue.edu (X.S.); gonz1140@purdue.edu (E.C.G.H.); chow85@purdue.edu (J.C.); fsanmartin@purdue.edu (F.S.M.-G.)
[2] School of Food and Agriculture, University of Maine, Orono, ME 04469, USA; qing.jin@maine.edu
*Correspondence: chen3370@purdue.edu.

Table S1. Operating costs applied in the study.

Item	Value
**Variable operating costs [a]**	
Canola meal ($/kg)	0.3
Alcalase ($/kg)	23
NaOH ($/kg)	0.45
HCl ($/kg)	0.22
Water ($/ton)	0.7
Electricity ($/kWh)	0.065
Steam ($/metric ton)	17
Cooling water ($/MT)	1
Solid waste disposal fee ($/kg)	0.06
**Fixed operating costs [b]** [1]	
Total labor number	6
Average labor salaries ($/year)	50,000
Labor burden ($/year)	90% of the labor salaries
Maintenance ($/year)	3% of inside-battery-limits equipment costs
Property insurance ($/year)	0.7% of fixed capital investment
Depreciation ($/year)	5% of total capital cost

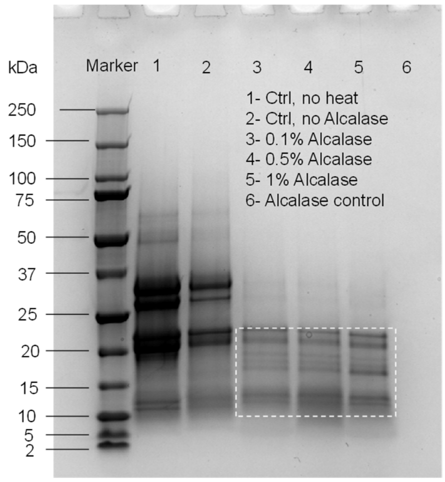

Figure S1. SDS-PAGE profile of canola meal proteins extracted with varying Alcalase concentration.

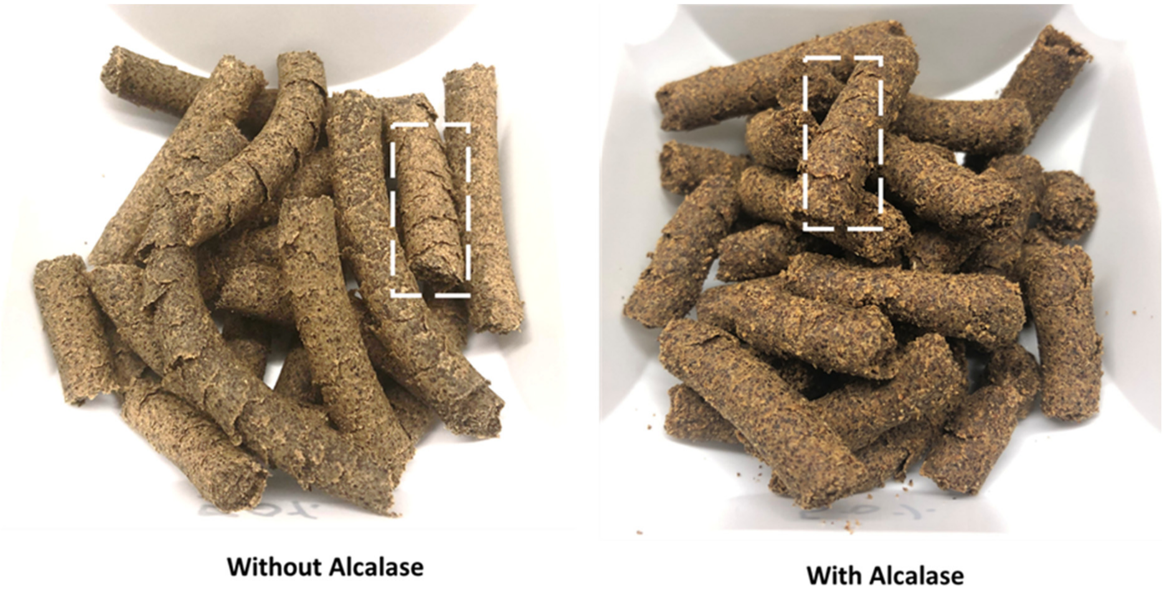

Figure S2. Enlarged photographs of canola meal extrudates at 55% moisture content without and with Alcalase.

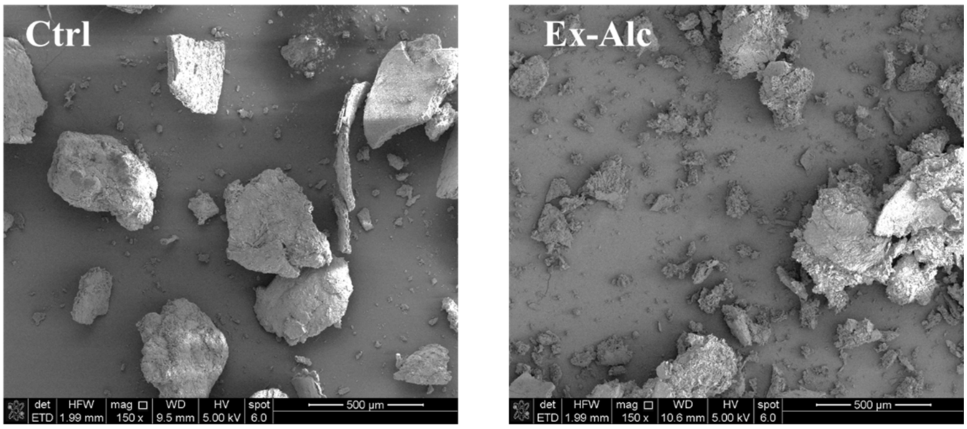

Figure S3. SEM micrographs of canola meal extracts of untreated control (Ctrl) and enzymatic reactive extrudates (Ex-Alc) at 65% moisture.

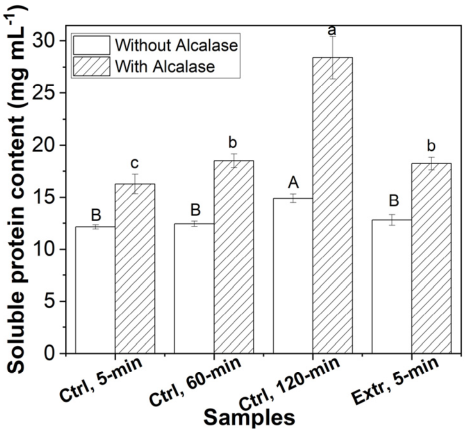

Figure S4. Soluble protein content of non-extruded controls (Ctrl) and extrudates (Extr) at 5, 60, and 120 min of alkaline extraction at pH 9, both without and with Alcalase. Different letters on top of the bars indicate significant differences among the data (P< 0.05).

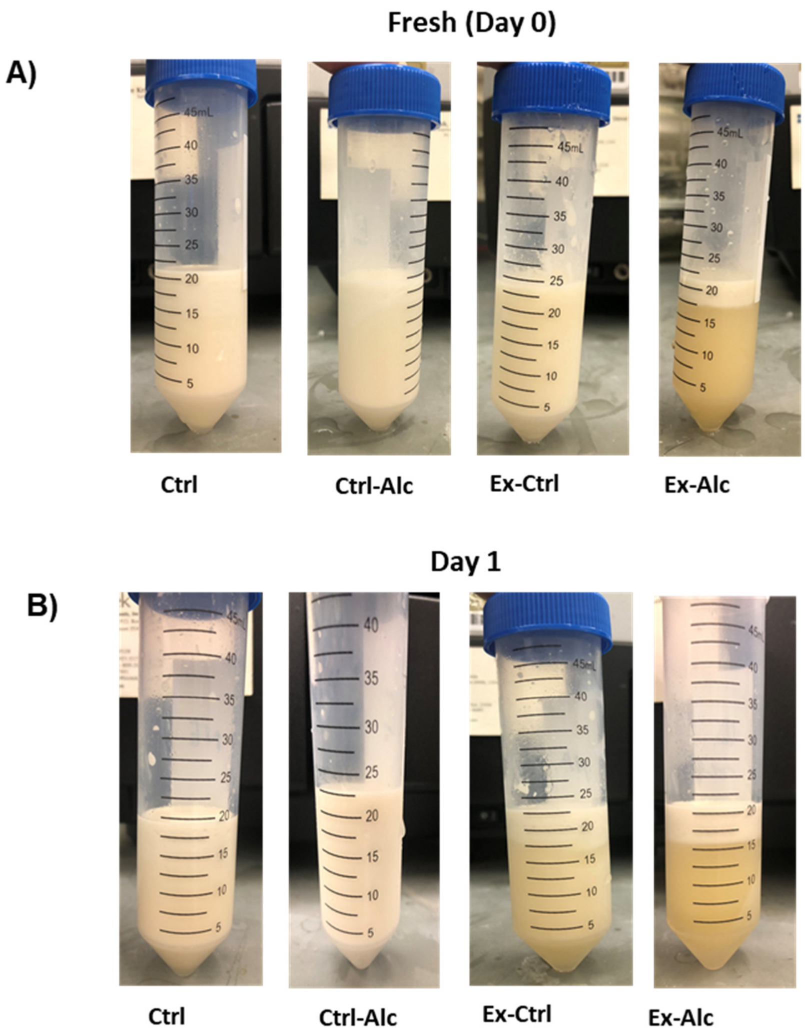

Figure S5. Photographs of emulsions formed with canola meal extracts at **(A)** day 0 **(B)** day 1. Ctrl, alkaline extraction without Alcalase; Ctrl-Alc, alkaline extraction with Alcalase; Ex-Ctrl, alkaline extraction of extruded samples without Alcalase; Ex-Alc, alkaline extraction of extruded samples with Alcalase

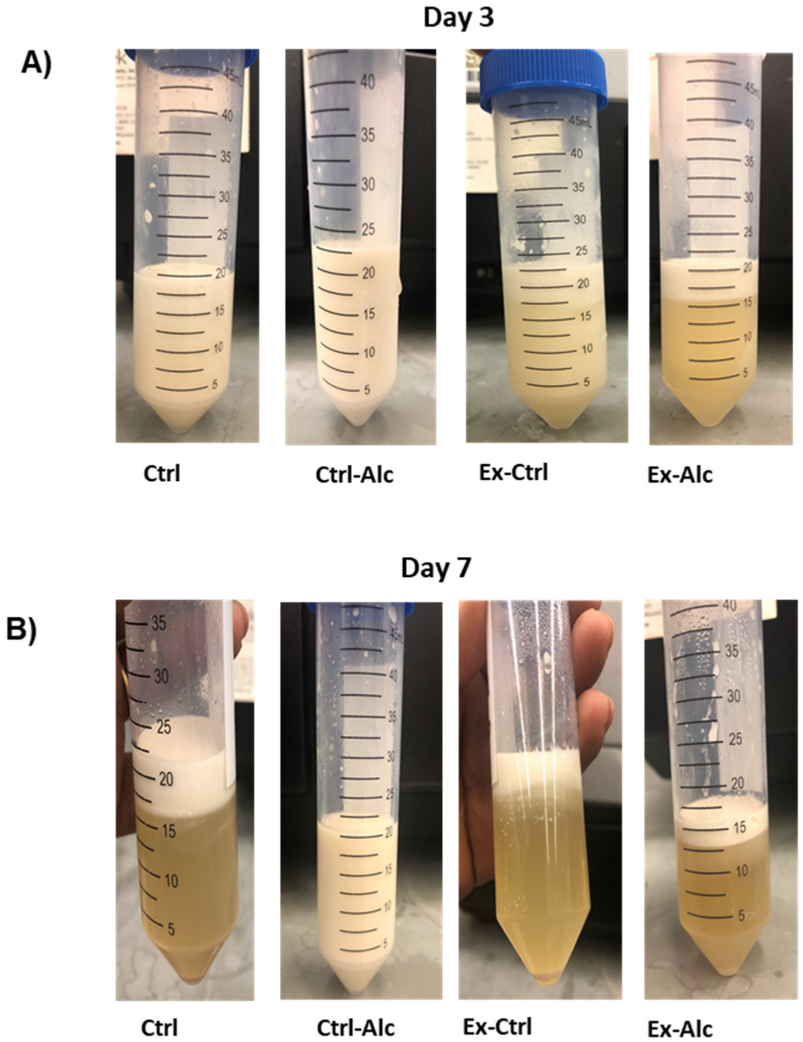

Figure S6. Photographs of emulsions formed with canola meal extracts at **(A)** day 3 **(B)** day 7. Ctrl, alkaline extraction without Alcalase; Ctrl-Alc, alkaline extraction with Alcalase; Ex-Ctrl, alkaline extraction of extruded samples without Alcalase; Ex-Alc, alkaline extraction of extruded samples with Alcalase

References

1. Humbird, D.; Davis, R.; Tao, L.; Kinchin, C.; Hsu, D.; Aden, A.; Schoen, P.; Lukas, J.; Olthof, B.; Worley, M.; et al. *Process Design and Economics for Biochemical Conversion of Lignocellulosic Biomass to Ethanol: Dilute-Acid Pretreatment and Enzymatic Hydrolysis of Corn Stover*; 2011; p. NREL/TP-5100-47764, 1013269. https://doi.org/10.2172/1013269.
